# Intravenous administration of silver nanoparticles causes organ toxicity through intracellular ROS-related loss of inter-endothelial junction

**DOI:** 10.1186/s12989-016-0133-9

**Published:** 2016-04-29

**Authors:** Hua Guo, Jing Zhang, Mary Boudreau, Jie Meng, Jun-jie Yin, Jian Liu, Haiyan Xu

**Affiliations:** 1 Institute of Basic Medical Sciences, Chinese Academy of Medical Sciences & Peking Union Medical College, Beijing, China; 2 National Center for Toxicological Research, US Food and Drug Administration, Jefferson, AR 72079 USA; 3 Center for Food Safety and Applied Nutrition, US Food and Drug Administration, College Park, MD 20740 USA

**Keywords:** Silver nanoparticles, Peripheral inflammation, ROS, Inter-endothelial junctions, Silver nitrate

## Abstract

**Background:**

Administration of silver nanoparticles (AgNPs) to mice could result in their distribution and accumulation in multiple organs, with notable prominence in liver, lungs, and kidneys. However, how AgNPs transport through blood vesicular system to reach the target organs is unclear, and the precise differences in the mechanisms of toxicity between AgNPs and silver ions still remain elusive. In the present research, the pathological changes on these target organs with a focus on inter-endothelial junction was investigated to gain a new insight of AgNPs toxicity by comparing the mechanisms of action of AgNPs and AgNO_3_.

**Methods:**

We investigated the in vitro cytotoxicity of either citrated-coated AgNPs (10, 75, and 110 nm) or silver nitrate (AgNO_3_) following 24 h incubations (1–40 μg/mL) in the presence of primary human umbilical vein endothelial cells (HUVEC). Meanwhile, we detected the effects of AgNPs on intercellular conjunction and intracellular ROS by VE-cadherin staining and 2′, 7′-dichlorodihydrofluorescein diacetate (DCFH-DA) assay, respectively. To assess in vivo toxicity, we administered single or multiple intravenous injections (25 μg Ag for AgNPs and 2.5 μg Ag for AgNO_3_ per dose) to mice.

**Results:**

In the in vitro study, the TEM observation showed that AgNPs were taken up by endothelial cells while AgNO_3_ was taken up little. Meanwhile AgNPs incubation induced the elevation of intracellular ROS and down-regulation of VE-cadherin between the endothelial cells and affected the cytoskeleton actin reorganization, which could be rescued by antioxidant *N*-acetylcysteine. In contrast, AgNO_3_ caused direct cell death when the concentration was higher than 20 μg/mL and without ROS induction at lower concentration. The release of AgNPs from leaking vessels induced peripheral inflammation in the liver, lungs, and kidneys, and the severity increased in proportion to the diameter of the AgNPs used.

**Conclusion:**

It is AgNPs but not AgNO_3_ that were taken up by vascular endothelial cells and induced intracellular ROS elevated, which was closely related to disruption of the integrity of endothelial layer. The AgNPs-induced leakiness of endothelial cells could mediate the common peripheral inflammation in liver, kidney and lung through intravenous exposure.

**Electronic supplementary material:**

The online version of this article (doi:10.1186/s12989-016-0133-9) contains supplementary material, which is available to authorized users.

## Background

In the past decade AgNPs have attracted intense interests from academia and industry due to their more potent antibacterial activity than that of conventional silver compounds. The list of potential applications for AgNPs is widespread and includes various house wares, electronics, and health care devices [1–6]. Meanwhile, the prospect for increased exposure of humans and other organisms to AgNPs has generated discussions focused on the safety and public health impact of future products that may contain AgNPs. The toxicological effects of AgNPs on the health have been rigorously investigated both in vitro and in vivo, and these studies are nicely summarized in several comprehensive reviews [7–13]. While numerous studies have shown that the toxicological characteristics of AgNPs differ from those of silver ions, the precise differences in the mechanisms of toxicity between AgNPs and silver ions remain elusive [14–16]. Our goal was to gain a better understanding of AgNPs toxicity by comparing the mechanisms of action of AgNPs and AgNO_3_ of both in vitro studies in HUVEC cell lines and in vivo studies in mice. We hypothesized that AgNPs and AgNO_3_ may exert differential effects within the blood vessel wall of normal, blood-filled vessels. Some investigators have reported that when AgNPs are administered intravenously to mice [17–21] or rats [22–28], those particles become distributed to most major organs, particularly to the spleen, liver, lungs, and kidneys [17, 20, 23, 26, 28, 29]. This strongly suggests these organs the most likely to be affected by any toxic effects of AgNPs, which have been reported to include oxidative DNA damage and apoptosis in the liver [17, 18] and alveolar wall thickening and the infiltration of focal inflammatory cells in the lungs [20]. Comparative toxicity studies of AgNPs in the blood vessels are rarely reported; moreover, the mechanisms underlying the transport of AgNPs through the walls of blood vessels, a necessary condition for subsequent organ toxicity, have not been well characterized.

In this study, we evaluated the effects of three different diameters of AgNPs and AgNO_3_ on in vitro cytotoxicity in HUVEC and in vivo toxicity to the blood vessel walls of the liver, kidneys, and lungs of Balb/c mice following intravenous administration. We show that AgNPs, but not AgNO_3_, induced the significant elevation of intracellular ROS and caused loss of the conjunction between endothelial cells, which not only allowed AgNPs to overcome the barrier of the endothelial layer and accumulate in the liver, lung and kidney, but also resulted in peripheral inflammation in these organs.

## Results

### Characterizations of AgNPs

**Fig. 1:**
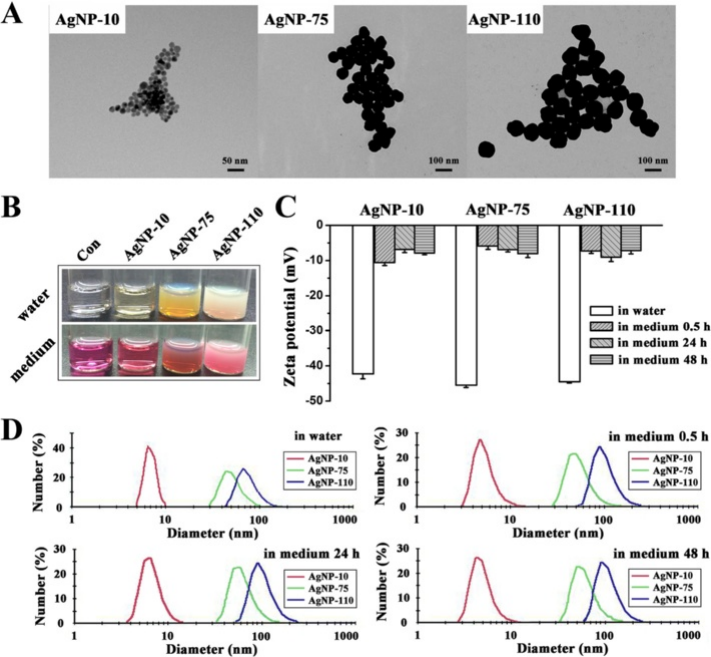
Characterizations of AgNPs. **a** The morphology of AgNPs by TEM observation. **b** Solution samples of AgNPs in pure water (*up*) and in the serum-containing culture medium (*bottom*). **c** The Zeta potential value of AgNPs in water and in the serum-containing culture medium for different incubation time. **d** The dynamic size distribution of AgNPs in water and in the serum-containing culture medium for different incubation time

TEM micrographs show that the three sizes of AgNPs are homogeneously spherical, and the average diameter for AgNP-10, AgNP-75 and AgNP-110 was determined as 11 ± 1 nm, 76 ± 6 nm and 107 ± 8 nm from the statistic counting of 100 nanoparticles respectively (Fig. 1a). The AgNPs were equally well dispersed in both water and in cell culture medium containing 10 % serum even after 48 h (Fig. 1b–d). The surfaces of AgNPs are negatively charged as −42.3 ± 1.4 mV, −45.5 ± 0.7 mV and −44.5 ± 0.4 mV for AgNP-10, AgNP-75 and AgNP-110 respectively in water, and became −7.9 ± 0.5 mV, −8.0 ± 1.1 mV and −7.2 ± 1.0 mV respectively in the serum-containing culture medium after 48 h incubation (Fig. 1c). The average hydrodynamic diameter for AgNP-10, AgNP-75 and AgNP-110 determined by DLS was 6.2 ± 1.6 nm, 72.0 ± 0.9 nm and 99.3 ± 1.7 nm respectively in water, and 12.6 ± 3.9 nm, 104.6 ± 0.2 nm and 147.0 ± 1.7 nm respectively in the serum-containing culture medium after 48 h incubation. The size distribution for each kind of AgNPs is consistent to Guassian distribution approximately no matter in water or in the cell culture medium (Fig. 1d). The decrease of Zeta potential and increase of AgNPs size in the serum-containing culture medium were attributable to the adsorption of serum components onto the AgNPs surface [30].

### Intravenously injected AgNPs cause peripheral inflammation

**Fig. 2:**
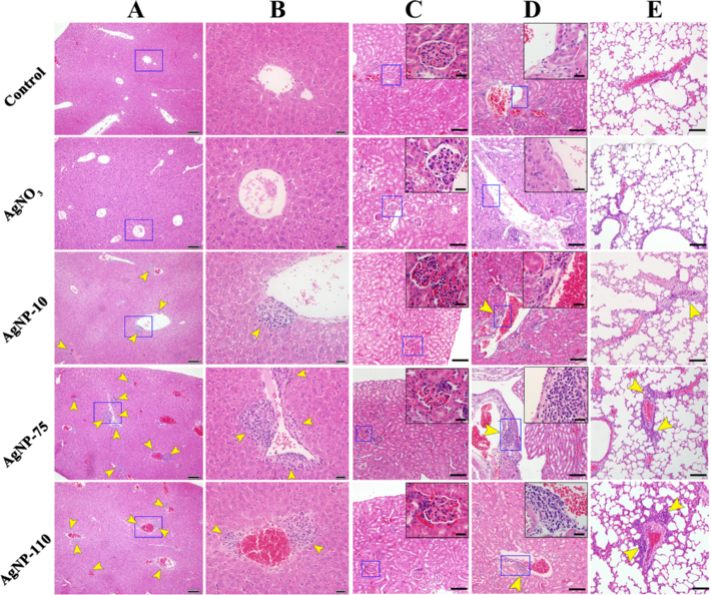
Histological and pathological observations for mice received multi-dose administration of AgNPs or AgNO_3_. Mice were injected with 25 μg AgNPs or AgNO_3_ solution containing 2.5 μg Ag on Day 1, 4 and 10. Tissue samples of liver, kidney, and lung were collected on the day 7 after the last injection. **a** The representative image of liver. The scale bar in the image represents 100 μm. **b** Magnification of **a**. The scale bar represents 20 μm. **c**-**d** Morphological observation of kidney. **e** H&E staining of lung. The inserted image in each image is the magnified area of the blue boxes in the rectangle; the short scale bar is 20 μm. The long scale bar represents 100 μm. Yellow arrow heads point to inflammatory cells around the vessels

Pathological and histological observations revealed remarkable differences between the control and AgNPs-treated mice that received multi-dose injections. In the organs of AgNPs-treated mice collected seven days after administration of the final dose, inflammatory infiltrates were detected in the vessel walls of liver (Fig. 2a–b), kidney (Fig. 2c–d), and lung (Fig. 2e). The surrounding inflammatory infiltrates (indicated by yellow arrows) mainly consisted of monocytes and neutrophils (Additional file: 1 Figure S2). These cells are generally accepted as recruited from the circulating blood, suggesting that some invading substance(s) induced inflammatory reactions that then resulted in peripheral inflammation. The average numbers of infiltrates around hepatic vessels were counted to be 7, 21.5 and 18 for AgNP-10, AgNP-75 and AgNP-110, respectively. This pattern suggests that the degree of AgNPs-induced inflammation was associated with particle size. The severity of liver inflammation was greater in those mice treated with AgNP-75 and AgNP-110 than that observed in the livers of mice treated with AgNP-10. The inflammation degree over the slides was scored in blind. It was indicated that there was moderate inflammation in the liver for mice received injection of AgNP-75 and AgNP-110, and mild inflammation for mice received injection of AgNP-10. When further comparing inflammatory cells in the infiltrates, it was shown that the percentage of monocytes (indicated by green arrows) in the peripheral inflammation was 96.0, 95.4 and 93.7 % for AgNP-10, AgNP-75 and AgNP-110, while the percentage of neutrophils (indicated by red arrows) was 4.0, 4.6 and 6.3 %, but no statistical difference existing, which indicated that the different size of AgNPs induced the same characteristics of inflammatory reactions in the peripheral vessel and suggested a chronic inflammation process.

Although AgNPs also induced peripheral inflammation in kidney and lung tissues, only a couple of infiltrates were seen in each section, without significant difference among the size of AgNPs. In addition, other damages occurred in all three organs of interest: basement membrane injury was observed in glomeruli; pale stained cytoplasm was observed in the liver; lung tissues showed thickened alveolar and vessel walls.

**Fig. 3:**
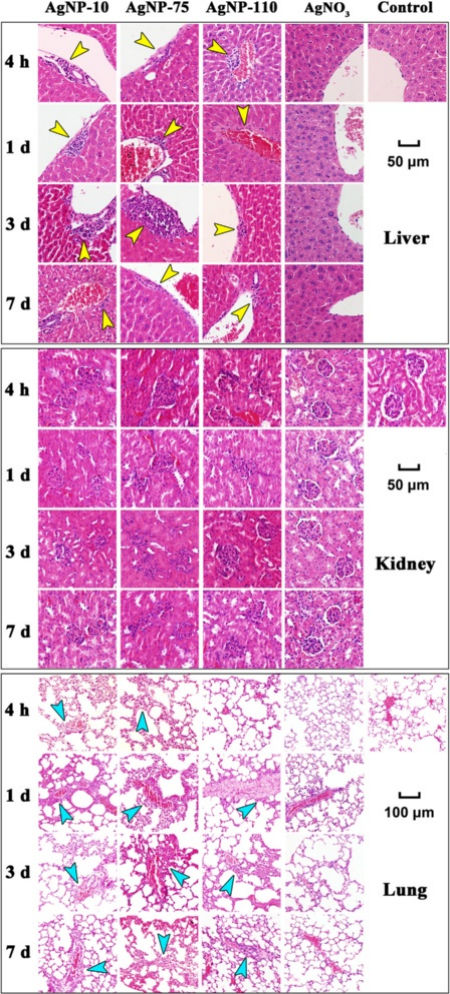
Pathological observations for mice received single-dose administration of AgNPs in different diameter or AgNO_3_. Mice were injected with 25 μg AgNPs or AgNO_3_ solution containing 2.5 μg Ag. Tissue samples of liver, kidney and lung were collected on 4 h, and 1, 3 or 7 days after the injection. Yellow arrow heads point to inflammatory cells around the hepatic vessels. Blue arrows point to slight fibrous hyperplasia around the blood vessels in the lung

Injection of a single dose of AgNPs induced a less toxicity in liver and lung tissues than that induced by multi-dose injections. The surrounding inflammatory infiltrates were only observed in the liver (yellow arrows), while fibrous hyperplasia was observed in the vicinity of blood vessels in the lung (blue arrows) (Fig. 3). The toxic effect of AgNPs was time dependent. Inflammatory infiltrates were observed in the liver as early as 4 h after injection and the infiltration area increased with time; inflammation attained greatest severity on the day 3 post injection. Subsequently, the inflammation subsided, but inflammatory infiltrates remained present even seven days post injection. Interestingly, intravenous administration of AgNO_3_ did not cause appreciable peripheral inflammation (Fig. 3), shedding the light on the difference of AgNPs from silver ions in the toxicity and suggesting that Ag particulates have unique mechanisms that cause the peripheral inflammation.

We next took AgNP-110 for example to investigate the localization of AgNPs in the liver of mice after 1 h or 24 h of intravenous injection. TEM observations showed that AgNP-110 located nearby microvascular structures among hepatic cells (Additional file: 1 Figure S3). The AgNP-110 in the liver tissue became irregular in shape, implying AgNP-110 spontaneously dissolved due to the complex biological environment of liver. Above results suggest that the cellular uptake of AgNPs is the initiated step to induce peripheral inflammation that was contributed by both AgNPs themselves and Ag ions continuously dissolving from the AgNPs.

### AgNPs can be taken up by vascular endothelial cells and cause cell leakiness by reducing inter-endothelial junctions

**Fig. 4:**
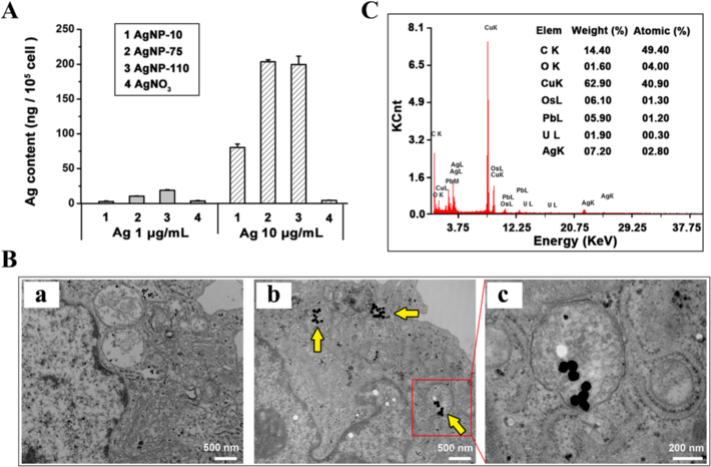
AgNPs uptake by HUVEC cells. **a** The intracellular silver content of cells after incubation with AgNPs or AgNO_3_ at different concentrations for 1 h. **b** TEM images of AgNP-75 (*yellow arrow heads pointed*) locating in the cells, in which (*a*) Control cells, (*b*) AgNP-75 treated cell, (*c*): magnification of (*b*), showing AgNP-75 in the vesicles. **c** Chemical composition analysis of (*b*) using energy-dispersive X-ray spectroscopy (EDX)

In order to determine the mechanism for the differential effects on blood vessels between AgNPs and AgNO_3_, in vitro studies were conducted with endothelial cells, since these cell types form the barrier layer in the vessel lumen. The results of ICP-MS analysis of endothelial cells for Ag content are shown in Fig. 4a. The intracellular Ag content of endothelial cells increased as the concentration of AgNPs was increased from 1 to 10 μg/ml; however, endothelial cells exposed to AgNP-75 and AgNP-110 demonstrated higher intracellular Ag content than cells exposed to AgNP-10. In contrast, only very low cellular content of Ag was detected in cells exposed to AgNO_3_ at 1 or 10 μg/mL of Ag. Taken an example of AgNP-75 incubated with endothelial cells for 24 h, TEM images show that there were round nanoscale particles in the cells and mainly located in autolysosomes and vesicles (Fig. 4b). Further examination with energy-dispersive X-ray spectroscopy (EDX) proved that the particles accumulating in the cells were composed of silver (Fig. 4c), confirming that AgNPs were taken up by the cells.

**Fig. 5:**
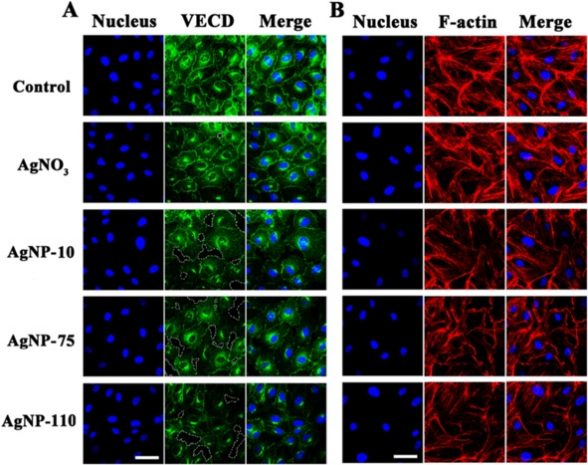
The reduction of inter-endothelial junction and cytoskeleton actin of cells exposed to AgNPs or AgNO_3_. Cells were incubated for 1 h with AgNP-10, AgNP-75, AgNP-110 or AgNO_3_ at 1 μg/mL of Ag. **a** Inter-endothelial junction stained with VE-cadherin antibody. **b** Cytoskeleton actin stained with tetramethyl rhodamine-conjugated phalloidin. The scale bar represents 20 μm

Next the intercellular junction protein, VE-cadherin, and cytoskeleton actin fibers of HUVEC cells were investigated to evaluate the effect of AgNPs and AgNO_3_ on the integrity of the vascular endothelial layer. As shown in Fig. 5a, endothelial cells without any treatment arrayed neatly and showed clear and dense VE-cadherin in the edge of cells. After treatment with AgNPs (1 μg/mL) for 1 h, gaps appeared among the cells and the distribution of VE-cadherin became faint and discontinuous. Along with the reduction of intercellular endothelial junction protein, cytoskeleton actin fibers became shorter (Fig. 5b).

### AgNPs-induced intracellular ROS elevation contributes long-term organ toxicity

**Fig. 6:**
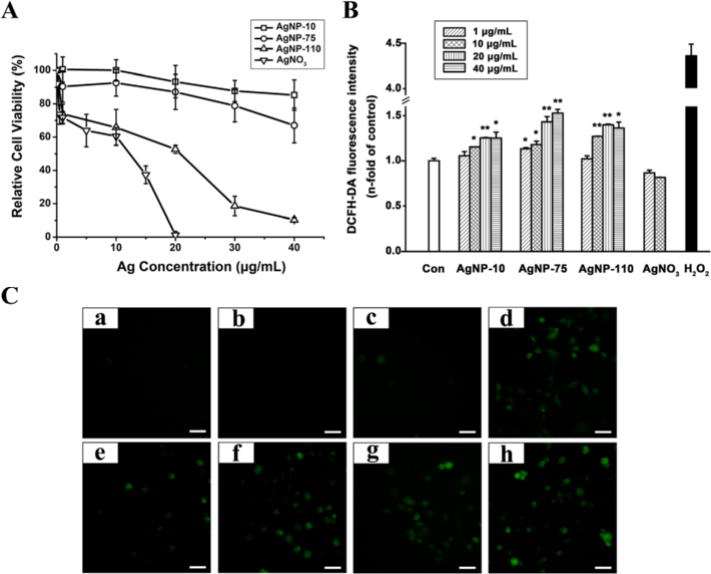
The viability and intracellular ROS of cells exposed with AgNPs or AgNO_3_. **a** Cell viability from CCK-8 assay. **b** The intracellular ROS level caused by AgNPs or AgNO_3_ exposure for 1 h. The H_2_O_2_ group was set as positive control. The * represents significant difference between control group and AgNP-75 treated group (*: *p* < 0.05, **: *p* < 0.01). **c** Representative fluorescence images of cells stained by DCFH-DA, in which (*a*) control group, (*b* and *c*) cells incubated with AgNO_3_ at 1 μg/mL and 10 μg/mL of Ag, (*d*) cells exposed to 7.5 mg/mL H_2_O_2_, (*e*-*h*) cells treated with 1, 10, 20 and 40 μg/mL AgNP-75. The scale bar represents 50 μm

Data from CCK-8 assay indicate that AgNPs inhibited cell viability in concentration and size dependent manners as shown in Fig. 6a. It should be noted that there are differences in the inhibitory behavior between AgNPs and AgNO_3_. Cells exposed to AgNO_3_ showed a steeper decrease in viability as Ag concentrations were increased. In contrast, the viability of cells exposed with AgNP-10 and AgNP-75 was decreased only 5 and 10 % respectively when Ag concentration was increased to 20 μg/mL, and gradually decreased to 10 and 30 % respectively when Ag concentration was increased to 40 μg/mL. The toxic effect of AgNP-110 on HUVEC cells was more serious than that of AgNP-10 and AgNP-75, but slighter less than that of AgNO_3_. Furthermore, Hoechst/PI assay was employed to compare the cell death induced by AgNPs and AgNO_3_. It was shown that cells treated with AgNO_3_ at 10 μg/mL of Ag for 20 min were stained by both red and bright blue fluorescence, indicating the cells were occurring necrosis in the short term of AgNO_3_ exposure; meanwhile, cells treated with AgNP-110 at 10 μg/mL for 40 min were just stained by light blue fluorescence, indicating the cells were undergoing early apoptosis (Additional file: 1 Figure S4). These data suggested that AgNO_3_ induced cells death through a mechanism different from AgNPs. On the basis of above results and previous literature report [31], we would suggest that AgNO_3_ is likely to induce cell death by inducing membrane damage, which was different from AgNPs that were largely taken up by the cells and thus induced cell apoptosis.

It has been demonstrated that intracellular ROS contributes to barrier dysfunction in endothelial cells along the vessel lumen [32]. The loss of cell-cell adhesion occurred in parallel to and is dependent upon the intracellular production of ROS [33]. The different inhibitory behaviors of AgNPs and their effect on the integrity of endothelial cells prompted the examination of ROS production in endothelial cells exposed to AgNPs or AgNO_3_ using the probe of 2′, 7′-dichlorodihydrofluorescein diacetate. Results show that the level of intracellular ROS was elevated in the cells incubated with AgNPs in a concentration dependent manner (Fig. 6b–c); however, cells exposed to AgNO_3_ did not produce ROS to any significant extent when the concentration reached 10 μg/mL of Ag, and higher concentration of AgNO_3_ caused cell death directly. In order to verify whether AgNPs interfered the quantitative results of ROS, the fluorescence intensity of the cells treated with AgNPs in the absence of DCFH-DA and the fluorescence intensity generated by AgNPs interacting with DCFH-DA (in absence of cells) were examined, which only accounted for 1.1 and 2.7 % in that produced by cells treated with AgNPs in the presence of DCFH-DA. Taken results above it is suggested that silver ions caused acute cell death due to the direct interaction with cell membrane, while AgNPs affected cells viability within longer term.

**Fig. 7:**
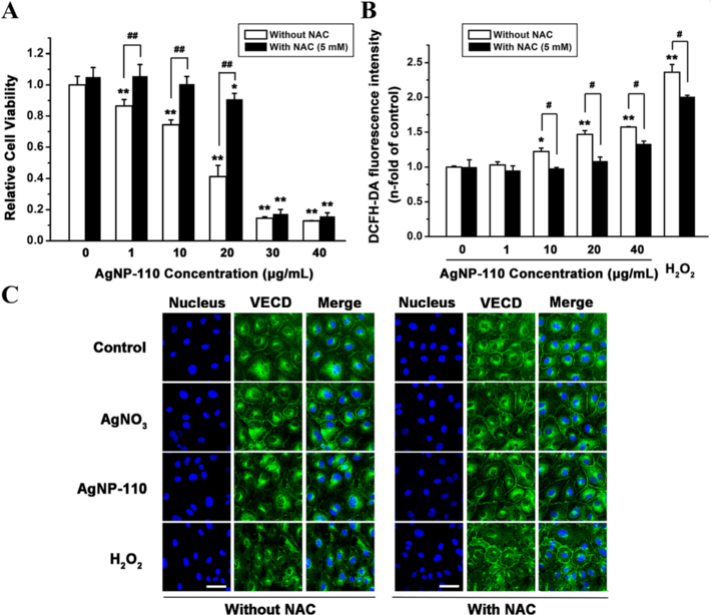
Rescue effects of NAC against treatment with AgNP-110 at different concentrations on HUVEC cells. In NAC treated groups, cells were pretreated with 5 mM NAC for 1 h. **a** Cell viability from CCK-8 assay. **b** The intracellular ROS level. **c** Cells were incubated for 1 h with AgNP-110 or AgNO_3_ containing 1 μg/mL of Ag, inter-endothelial junction stained with VE-cadherin antibody. The scale bar represents 20 μm. The cells treated with 7.5 mg/mL H_2_O_2_ was set as positive control. The * represents significant difference between control group and AgNP-110-treated group (*: *p* < 0.05, **: *p* < 0.01), the # represents significant difference between groups with and without NAC pretreatment (#: *p* < 0.05, ##: *p* < 0.01)

To further explore the relation between intracellular ROS elevation and endothelial cells viability and VE-cadherin expression, the antioxidant NAC was used in rescue experiments, taking AgNP-110 as a model material. The NAC-pretreated cells exhibited higher viability than those without NAC pretreatment (Fig. 7a). Meanwhile, the level of ROS in NAC-pretreated cells was significantly lower than that in cells without NAC pretreatment when AgNPs exposure concentration ranged from 1 to 40 μg/mL (Fig. 7b). It is noticed that in Fig. 7b, the ROS level showed elevated tendency as AgNPs concentration increased both in the group of NAC-pretreatment (black column) or without NAC pretreatment (white column), however no significant difference was observed between that of control group and AgNP-110 group at 1 μg/mL, which might be due to the limited detecting sensitivity of DCFH-DA assay, because control cells would exhibit high background fluorescence [34]. Whether oxidative stress is the first event for AgNPs toxicity requires further investigations. Furthermore, significant rescue effect of NAC on the integrity of the vascular endothelial layer was observed when endothelial cells were exposed to AgNPs (Fig. 7c), gaps among the cells were few and the distribution of VE-cadherin was well-organized. These together imply that it is the nanoscale particles of silver that cause the reduction of inter-endothelial junction, affecting the integrity of the endothelial layer. In contrast, AgNO_3_ exposure at 1 μg/mL of Ag did not affect the expression of VE-cadherin, however higher concentration of AgNO_3_ induced immediate cell death. These data indicated the close relation between the AgNPs-induced intracellular ROS and the reduction of VE-cadherin.

## Discussion

The present study showed that one different biological effect between AgNPs and AgNO_3_ is the endocytosis of AgNPs but not AgNO_3_, evidenced by the result of ICP-MS measurement and TEM observation. After being internalized, AgNPs would interact with cells directly or dissolve Ag ions to interact with biological systems. As pointed out by George et al. [35], the oxygen radical production by AgNPs could be related to the reactive surface of nanomaterials or mediated by AgNPs direct interaction with cellular component. It has been reported that AgNPs induced ROS production by mitochondria, and increased mitochondrial ROS levels, causing the induction of apoptosis through the downstream signaling pathways [30, 36]. De Matteis et al. [37] revealed that AgNPs toxicity was mainly dependent on the presence of Ag ions inside cell cytosol. However, AgNO_3_-mediated cell death in the cell medium was inducement of impairment of cell membrane permeability to ions K^+^ and Na^+^ [38], which was independent of intracellular effects of AgNPs. As demonstrated in the present study, AgNPs can be taken up by endothelial cells and induced concentration-dependent intracellular ROS elevation, which could be rescued by antioxidant *N*-acetylcysteine (NAC) (Fig. 7). It is rational to infer that the elevation of intracellular ROS was resulted from the internalized AgNPs and closely related to the reduction of inter-endothelial junctions and the peripheral inflammation.

On the other hand, our findings showed that AgNO_3_ is hardly to be taken up by the cells and only induced a low and constant intracellular ROS level, though the acute cytotoxicity was observed, suggesting that direct exposure of Ag ions had other cytotoxic mechanism independent of elevating intracellular ROS. Other research groups also reported that the exposure of AgNO_3_ induced much lower ROS than AgNPs upon the cells [39], which supports our observation. In addition, in the in vivo experiments, the effective concentration of AgNPs or AgNO_3_ in blood was too low to induce vascular endothelial cells death directly due to the dilution effect of blood. However, AgNPs contacting the vessel wall could be taken up by the vascular endothelial cells to induce intracellular ROS, which was evidenced by TEM observation after intravenous exposure of AgNPs to mice (Additional file: 1 Figure S3).

**Fig. 8:**
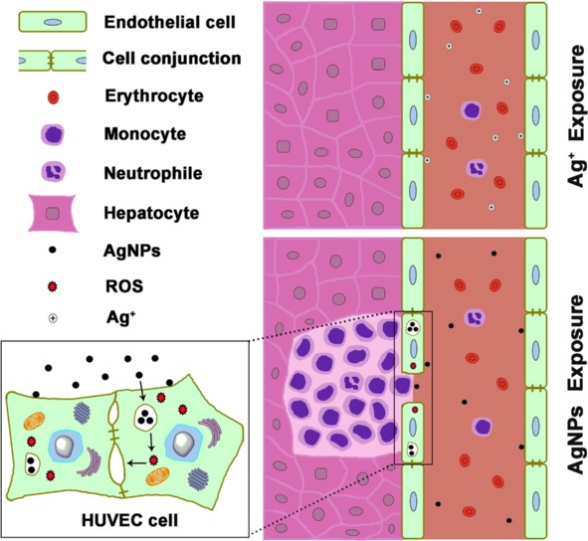
The intravenous exposure of AgNPs induced peripheral inflammation in liver. AgNPs in the circulating blood were taken up by vascular endothelial cells, which elevated the intracellular ROS level and reduced the conjunction between endothelial cells. The leakiness of endothelial cells resulted in the peripheral inflammation. On the contrary, little Ag ions were taken up by the cells

In the present study, we also observed that larger AgNPs caused more severity of liver inflammation (Fig. 2), which implied larger AgNPs could retain in cells longer and dissolve more Ag ions continuously, supporting the proposed mechanism. The ICP-MS results provided evidence that larger AgNPs were taken up more by endothelial cells, suggesting that the inflammation degree exerted by AgNPs dependent on the cellular uptake instead of direct exposure dose of AgNPs. One other group also observed that the toxicity of AgNPs in animals arises from the nanoparticulate identity [23]. Taking above together, the uptake of AgNPs by endothelial cells is the crucial factor that is responsible to the organ toxicity of AgNPs. Therefore, we would suggest the endocytosis of AgNPs is the initial and crucial step that accounts for the toxicity of AgNPs, which was illustrated schematically in Fig. 8.

The ROS elevation is associated with vascular endothelial cells detachment [32, 33]. VE-cadherin is a key molecule that maintains endothelial cell-cell integrity. It was reported that the VE-cadherin was lost when endothelial cells were exposed with TiO_2_ nanoparticles due to the direct combination of TiO_2_ nanomaterials to VE-cadherin, which caused leakiness of subcutaneous blood vessels in mice [40]. Results from our in vitro experiments showed that AgNPs also reduced the VE-cadherin between endothelial cells and disrupted endothelial conjunction (Fig. 5), while addition of antioxidant NAC reduced the disruption of endothelial conjunction, indicating that the elevation of intracellular ROS contributed to the reduction of VE-cadherin (Fig. 7). Nevertheless, we would consider that the mechanism of direct combination reported by Setyawati et al. [40] may also contribute to the reduction of endothelium junctions. Through this mechanism, AgNPs in the circulating blood leak out of vessels through the membrane damage, while AgNO_3_ has little chance to penetrate the vessel wall. The AgNPs leaking out of vessels would attract immune cells in the peripheral tissue, causing inflammatory reactions. Meanwhile, AgNPs entering into the peripheral tissue can dissolve Ag ions constantly [41], resulting local high concentration of Ag ions to induce the oxidative damage on the membrane of cells. Both together induced inflammatory infiltration around the vessels. Furthermore, our findings hinted that nanoparticles might have the common property of elevating the intracellular ROS level, however, depending on their size and chemical composition. It is an important issue that worth to conduct further investigations.

Taking above together, it is the particulate property and chemical activity of silver that allow AgNPs to overcome the barrier of endothelial layer to enter into peripheral tissue of vessels in the liver, kidney and lung, inducing immunological reactions and resulting in the peripheral inflammation in the organs. These results also explain why AgNPs were reported as predominantly accumulating in organs where plenty of vascular networks exist. Therefore, manufacturers should be aware that there may be additional safety considerations for products resulting in exposure to AgNPs that enter circulating blood directly from the veins or through the mucosa, damaged skin, or severe trauma.

## Conclusion

In summary, the uptake of AgNPs by endothelial cells was the crucial step and accounted for the toxicity of AgNPs exposed intravenously, while the direct exposure of AgNO_3_ was taken up little. Intravenously injected AgNPs could overcome the barrier of endothelial layer through reducing the interaction between VE-cadherin of endothelial cells, which induced leakiness of endothelial cells and resulted in the common peripheral inflammation in liver, kidney and lung.

## Methods

### NPs, cell stocks, and lab animals

Three sizes of citrate-coated AgNPs (having diameters of 10, 75, and 110 nm, and of 1.0 mg/mL; the nominal Ag concentration in 2 mM sodium citrate buffer, the percentage of ionic Ag is less than 0.02 % as described by the manufacturer) were purchased from nanoComposix (San Diego, CA), and were filter-sterilized before use. The filter-sterilized stocking solution of different diameters of AgNPs was examined with limulus reagent (Genscript biotechnology company) for quantification of LPS. Results showed the absorbance of AgNPs products was below the detection limit. AgNO_3_ (0.1 M standard solution, sterile) was purchased from Aladdin Industrial Corporation (Shanghai, China).

Primary human umbilical vein endothelial cells (HUVEC), endothelial cell medium (ECM), and endothelial cell growth supplements were all purchased from ScienCell Research Laboratories (San Diego, CA). These cells were grown to attachment on cell culture plates coated with 1 μg/cm^2^ Poly-L-Lysine and cultured at 37 °C in a humidified incubator with 5 % CO_2_. The ECM was supplemented with 5 % fetal bovine serum (FBS), penicillin/streptomycin and other endothelial cell growth supplements.

All the animal experiments reported herein were carried out in accordance with a protocol that was approved by the Institutional Animal Care and Use committee (Institute of Basic Medical Sciences, Chinese Academy of Medical Sciences, and Peking Union Medical College, Beijing, China). Female Balb/c mice (6–7 weeks of age) were maintained at the institutional experimental animal center under specific pathogen-free conditions. The mice were fed with autoclaved water and rodent chow pellets.

### Characteristics of AgNPs

#### Morphology study

We observed the shape of the AgNPs using a JEM-1400 plus transmission electron microscope (TEM, JEOL Ltd., Tokyo, Japan). Aqueous solutions of AgNPs (5 μg/mL) were dropped (~5 μL) on a copper grid, and then dried under ambient conditions. Resulting samples were imaged on the TEM attached to a CCD camera (Veleta, Olympus Soft Imaging Solutions GmbH).

#### Particle size and surface charge measurement

The hydrodynamic diameter and Zeta potential of particles in the AgNPs suspensions were determined by dynamic light scattering (DLS) and electrophoretic light scattering (ELS) analysis, respectively, using a ZetasizerNano ZS90 analyzer (Malvern Instruments Ltd, Malvern, UK). Briefly, stock solutions of AgNPs were dispersed in 1 mL of double-distilled water (DDW) or serum-containing medium following the incubation for 30 min, 24 h or 48 h. The appropriate level of laser power and the measurement position within the curette were determined automatically by the instrument. All measurements were carried out at room temperature.

### Cell viability assay

The viability of HUVEC incubated with AgNPs was determined using a cell count kit (CCK-8, Dojindo Molecular Technologies, Inc.) in accordance with the manufacturer’s protocol as follows: HUVEC cells were seeded into each well of 96-well cell culture plates at a density of 1 × 10^4^ cells/well and cultured overnight for adherence. The concentrations of AgNPs and AgNO_3_ were adjusted to yield final silver concentrations that ranged from 1 to 40 μg/mL, which was corresponding to the concentrations of particle number as 0.3–12 nM for AgNP-10, 0.7–28 pM for AgNP-75, and 0.2–9 pM for AgNP-110. For the rescue experiment, cells were treated with 5 mM of *N*-acetylcysteine (NAC; Sigma-Aldrich, USA) for 1 h prior to the exposure of AgNP-110. After incubation for 24 h at 37 °C, the cells were washed twice with PBS and incubated for an additional 2 h with fresh medium containing 10 μL of CCK-8 reagent. Then the absorbance of medium was measured at 450 nm using the Synergy H1 Hybrid Multi-Mode Microplate Reader (BioTek Instruments, Winooski, VT, USA). All measurements were performed in quadruplicate and the relative cell viability was expressed as percentage of controls that were cultured in the absence of AgNPs or AgNO_3_.

### Hoechst and PI staining

Hoechst 33342 and propidium iodide (PI) dyes (Beyotime Institute of Biotechnology, China) were used to evaluate the occurrence of apoptosis and necrosis mediated by AgNPs-110 or AgNO_3_. HUVEC cells were seeded at a density of 8 × 10^4^ cells/well in 24-well cell culture plates and incubated overnight for adhesion. Cells were then incubated with AgNP-110 for 40 min or AgNO_3_ for 20 min at 10 μg/mL of Ag. After washing with PBS for twice, the cells were incubated with 1.5 μL Hoechst 33342 and 1.5 μL PI in 300 μL cold buffer at 37 °C for 25 min. Subsequently, the cells were washed twice with PBS and then fixed with 4 % paraformaldehyde/PBS for 12 min at 37 °C. After rinsing with PBS, cells were mounted with an aqueous mounting medium. Micrographs were taken by the fluorescent microscope (Olympus, Tokyo, Japan).

### Immunostaining and laser confocal microscopy assay

In order to examine the effect of AgNPs on cell junctions, HUVEC cells were seeded at a density of 1 × 10^5^ cells/well in 24-well cell culture plates and incubated overnight for adhesion. Cells were then incubated with AgNP-10, AgNP-75, AgNP-110 or AgNO_3_ in 400 μL culture medium for 1 h at 1 μg/mL of Ag. For the rescue experiment, cells were treated with NAC (5 mM) for 1 h prior to the exposure of AgNP-110. Positive control cells were treated with 7.5 mg/mL hydrogen peroxide (H_2_O_2_) in the absence or presence of NAC treatment. Cells were then fixed with 4 % paraformaldehyde for 30 min at 37 °C. After rinsing with fresh PBS, cells were incubated with PBS containing 2 % BSA for 1 h at room temperature. Thereafter, cells were labeled with VE-Cadherin XPTM Rabbit antibody (1:200 dilution; Cell Signaling Technology, Danvers, MA, USA) in 2 % BSA/PBS for 1 h at 37 °C. The cells were then washed twice with PBS and incubated for 1 h at room temperature with 5 μg/mL Alexa Fluor®488-labeled goat anti-rabbit antibody (Cell Signaling Technology). Cells were washed with PBS and mounted with an aqueous mounting medium containing DAPI (Zhongshan Goldenbridge biotechnology Co, China). Micrographs were taken by FluoView FV1000 confocal microscope (Olympus) and analyzed by FluoView software (Olympus). Cells cultured in the normal medium with neither AgNPs nor AgNO_3_ were set as control.

To examine cell cytoskeleton reorganization mediated by AgNPs, fixed HUVEC cells were permeabilized with 0.5 % Triton X-100 for 5 min at 4 °C. The cells were rinsed with PBS, incubated in PBS containing 1 % BSA for 5 min, labeled with tetramethyl rhodamine-conjugated phalloidin (1:500, Sigma-Aldrich, USA) for 40 min at 37 °C, and then washed with PBS containing 0.5 % Tween 20. The cells were mounted with an aqueous mounting medium containing DAPI and visualized with the confocal microscope as above.

### Cellular uptake and intracellular localization studies of AgNPs

HUVEC cells were seeded in 6-well plate at 3.2 × 10^5^ cells/well and incubated overnight for adhesion. The cells were incubated with AgNPs (10 nm, 75 nm, 110 nm) or AgNO_3_ at 1 and 10 μg/mL of Ag for 1 h at 37 °C. After the 1 h exposure period, cells were rinsed with PBS, detached with trypsin, and collected by centrifugation. The cells were then re-suspended in 500 μL PBS. The cell number in each group was determined using an automated cell counter (TC10™, Bio-RAD, Hercules, CA). The cell suspension was transferred into conical flasks and digested with the mixture of nitric acid (3 mL) and H_2_O_2_ (1 mL). The conical flasks were placed in boiling water bath until the volume of acid was reduced to 1 mL. The weight of the resulting solution was adjusted to 4 g, using 2 % (*w/w*) nitric acid. The total silver concentration in the solution was determined with XSERIES 2 Quadrupole ICP-MS instrument (ThermoFisher Scientific Inc., Franklin, MA). The cellular uptake amount of AgNPs or AgNO_3_ was expressed as mass of Ag per cell.

The intracellular localization of AgNPs was examined by TEM and EDX analysis. Briefly, HUVEC cells were seeded at a density of 1.5 × 10^6^ cells/dish in a 100 mm dish and incubated overnight for cell attachment. The cells were treated with AgNP-75 at 20 μg/mL and incubated for 24 h to allow AgNPs uptake. Cells were washed three times with PBS, scraped gently from the dishes, and fixed with 2.5 % glutaraldehyde (Fluka) for 1 h. The resulting cells were dehydrated gradually by alcohol and embedded in epon. Sections were then cut with an ultra-microtome (Leica EM UC7, Leica Microsystems, Germany) and placed on copper grids for observation. AgNPs were identified by energy-dispersive X-ray spectroscopy (EDX, Tecnai G^2^ 20 S-TWIN TEM; FEI Company, USA).

### Intracellular ROS measurement

The intracellular ROS level was measured using the probe of 2′, 7′-dichlorodihydrofluorescein diacetate (DCFH-DA, Sigma-Aldrich, USA). HUVEC cells were seeded (8 × 10^4^ cells/well) in 24-well cell culture plates and incubated overnight for adhesion. The cells were treated with AgNP-10, AgNP-75 or AgNP-110 at 1 to 40 μg/mL, or AgNO_3_ at 1 to 20 μg/mL of Ag for 1 h at 37 °C, followed by washing the cells with PBS. For the cells treated with NAC and AgNPs, cells were treated with NAC (5 mM) for 1 h prior to the addition of different concentrations of AgNP-110. Serum-free medium (opti-MEM, Life Technologies, Grand Island, NY) containing 10 μM DCFH-DA was added to the plates for 30 min at 37 °C, the cells were then imaged by the fluorescent microscope (Olympus, Tokyo, Japan). After that the cells were washed with PBS, detached from the plates with trypsin, and collected by centrifugation. Positive control cells were treated with H_2_O_2_ in the absence or presence of NAC treatment. Flow cytometric analysis was conducted on the various cell treatment groups, using an excitation wavelength of 488 nm and emission wavelength of 525 nm (Accuri^TM^ C6 flow cytometer; BD Biosciences, San Jose, CA). In order to subtract the background resulting from AgNPs as well as the interaction of AgNPs with NAC, the fluorescence intensity of the cells treated with AgNPs in the absence of DCFH-DA and the fluorescence intensity generated by AgNPs interacting with DCFH-DA (in absence of cells) were examined.

### Intravenous administration of AgNPs in mice

#### Multi-dose injection

Female Balb/c mice were injected with 50 μL of 5 % isotonic glucose solution containing AgNP-10, AgNP-75, and AgNP-110 (25 μg Ag) or AgNO_3_ (2.5 μg Ag, positive control) [37, 42] on Day 1, 4 and 10. Each group consisted of 3 mice and a negative control group of 3 mice only received injections of 5 % glucose solution. Seven days after being injected with the final dose for the assigned treatment condition, all mice were sacrificed by cervical dislocation and organs were collected for pathological analysis.

#### Single-dose injection

AgNPs (10, 75, and 110 nm) solution containing 25 μg Ag or AgNO_3_ solution containing 2.5 μg Ag was injected intravenously. Mice were sacrificed at 4 h, and 1, 3 or 7 days post the injection (*n* = 3 for each group per time point). Organs were collected for the histopathological evaluation.

### Histopathological evaluation

The collected organs were rinsed in PBS, fixed in 4 % formaldehyde for one week, and then embedded in paraffin. Organ sections were processed and stained with haematoxylin and eosin (H&E staining) following standard procedures. The sections of liver, kidney and lung were visualized with light microscopy and the number of inflammatory infiltrates in peripheral of vessels was counted in five separate sections (BX53, Olympus, Tokyo, Japan) with a CCD camera (DP72; Olympus). The quantified characterization for infiltrated cells was performed through blinded histological observations. Inflammation cells in 12 infiltrates in the five tissue sections were identified. The inflammation degree over the slides was scored in blind by two pathologists in the Peking Union Medical College Hospital.

#### TEM observation of localization of AgNPs in liver tissue

AgNP-110 solution containing 25 μg Ag in 50 μL of 5 % isotonic glucose solution was intravenously injected to mice. After 1 h and 24 h post the injection, mice were anesthetized with intraperitoneal injection of 1 % pentobarbital sodium solution (Sigma-Aldrich, USA). Then liver tissues were collected and fixed with 2.5 % glutaraldehyde overnight. After being washed and post-fixed in 1 % OsO_4_ for 30 min, the specimens were dehydrated gradually by alcohol and embedded in epon. Sections were then cut with an ultra-microtome and placed on copper grids for observation.

### Statistical analysis

Values are shown as means ± SD for all treatment groups. All experiments except indicated were carried out in triplicate and the data were analyzed using Student’s t test to determine the statistical difference, followed by comparisons between the vehicle control and the individual treatment groups. *P*-values < 0.05 or, in some instances, <0.01 were considered statistically significant.

## Additional file

Additional file 1: Figure S1. Size and Zeta potential of AgNP-110 dispersed in water and 5 % glucose solution. **Figure S2.** Characterization of peripheral inflammation in liver tissues of mice received multi-dose administration of AgNPs. **Figure S3.** Localization of AgNP-110 in the liver of mice received intravenous administration of AgNP-110. **Figure S4.** Representative fluorescent images of cells stained by Hoechst/PI after exposure to AgNP-110 or AgNO_3_. (DOC 7779 kb)

## Abbreviations

AgNPs: Silver nanoparticles
DAPI: 4, 6-diamino-2-phenyl indole
DCFH-DA: 2′, 7′-dichlorodihydrofluorescein diacetate
DDW: Double-distilled water
DLS: Dynamic light scattering
EDX: Energy-dispersive X-ray spectroscopy
ELS: Electrophoretic light scattering
HUVEC: Human umbilical vein endothelial cells
ICP-MS: Inductively coupled plasma-mass spectrometry
NAC: *N*-acetylcysteine
PI: Propidium iodide
ROS: Reactive oxygen species
TEM: Transmission electron microscope
