# Supplementary material for: Intravenous administration of silver nanoparticles causes organ toxicity through intracellular ROS-related loss of inter-endothelial junction
Source: Part Fibre Toxicol. 2016 Apr 29;13:21. doi: 10.1186/s12989-016-0133-9 (PMC4850669; doi:10.1186/s12989-016-0133-9)
Supplement: Additional file 1: Figure S1. — Size and Zeta potential of AgNP-110 dispersed in water and 5 % glucose solution. Figure S2. Characterization of peripheral inflammation in liver tissues of mice received multi-dose administration of AgNPs. Figure S3. Localization of AgNP-110 in the liver of mice received intravenous administration of AgNP-110. Figure S4. Representative fluorescent images of cells stained by Hoechst/PI after exposure to AgNP-110 or AgNO3. (DOC 7779 kb) [file 12989_2016_133_MOESM1_ESM.doc]

**Intravenous administration of silver nanoparticles causes organ toxicity through intracellular ROS-related loss of inter-endothelial junction**

Hua Guo^1*^, Jing Zhang^1*^, Mary Boudreau^2^, Jie Meng^1^, Jun-jie Yin^3^, Jian Liu^1¶^, Haiyan Xu^1¶^

1 Institute of Basic Medical Sciences, Chinese Academy of Medical Sciences & Peking Union Medical College, Beijing, China.

2 National Center for ​Toxicological​ Research, US Food and Drug Administration, Jefferson, AR 72079, USA.

3 Center for Food Safety and Applied Nutrition, US Food and Drug Administration, College Park, MD 20740, USA.

^¶^ Corresponding author: Haiyan Xu, Jian Liu

^*^Theses authors contributed equally to this work.

List of author Email: Hua Guo [sarahguo0801@sina.cn](mailto:sarahguo0801@sina.cn) – Jing Zhang [zhjingyu@sina.cn](mailto:zhjingyu@sina.cn) – Mary Boudreau [mary.boudreau@fda.hhs.gov](mailto:mary.boudreau@fda.hhs.gov) – Jie Meng [mengjie@ibms.pumc.edu.cn](mailto:mengjie@ibms.pumc.edu.cn) – Jun-jie Yin – [junjie.yin@fda.hhs.gov](mailto:junjie.yin@fda.hhs.gov) – Jian Liu [liujian@ibms.pumc.edu.cn](mailto:liujian@ibms.pumc.edu.cn) – Haiyan Xu [xuhy@pumc.edu.cn](mailto:xuhy@pumc.edu.cn)


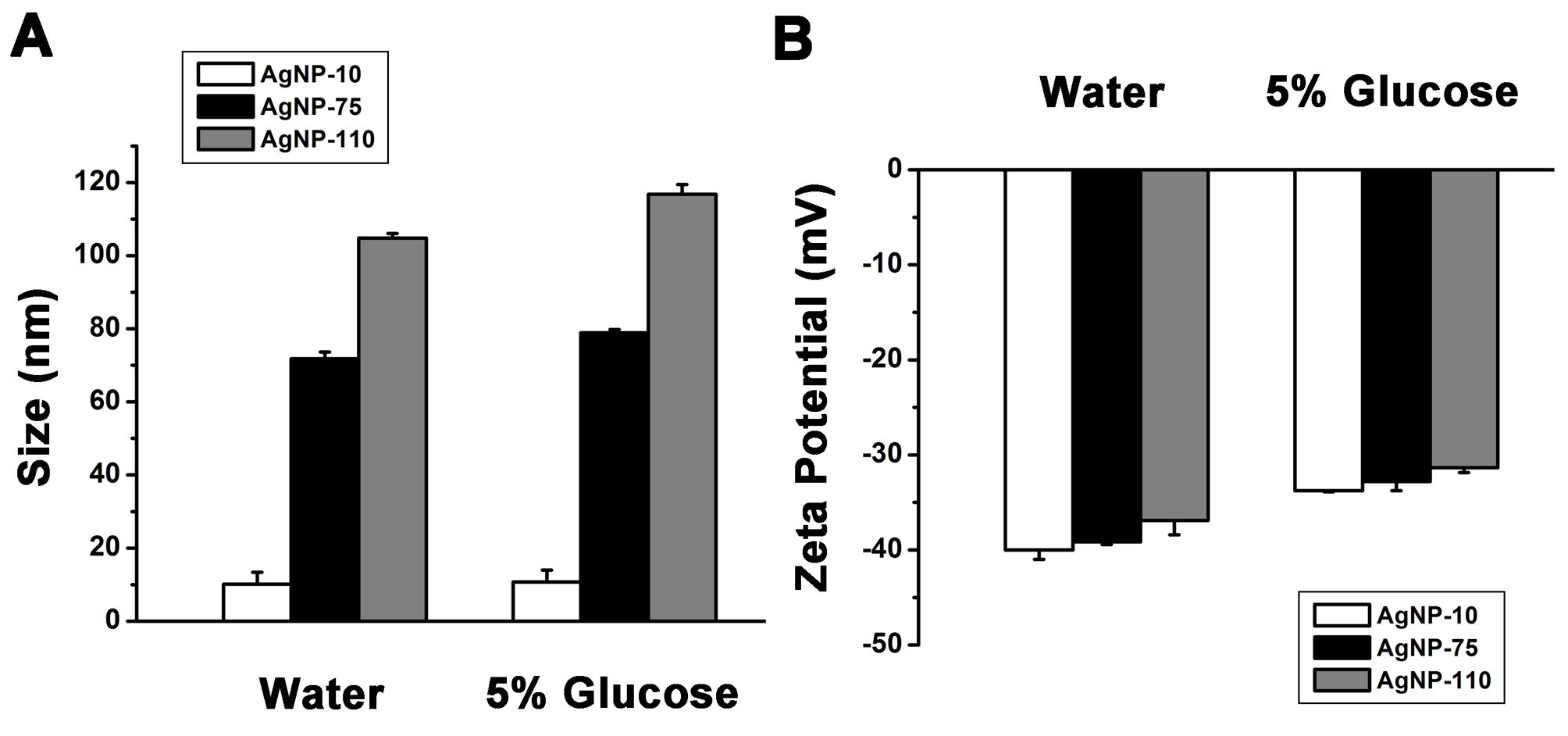


Supplementary Figure S1. Size and Zeta potential of AgNP-110 dispersed in water and 5% glucose solution.


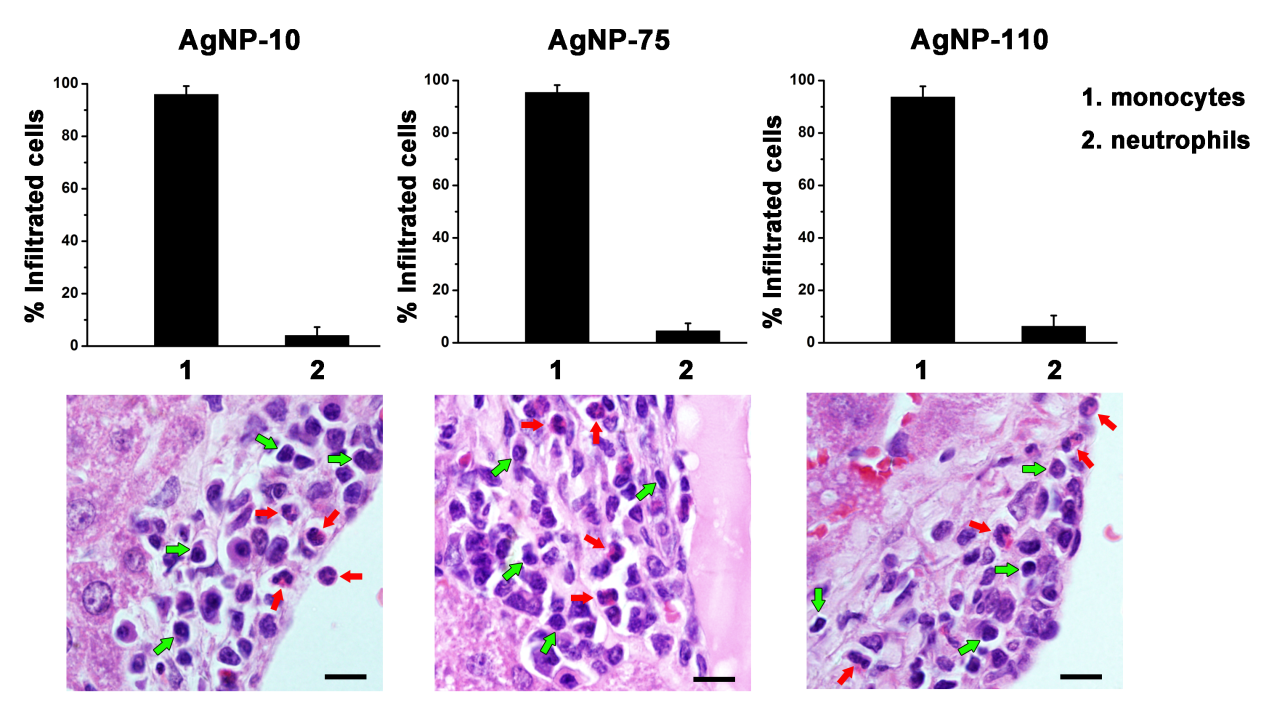


Supplementary Figure S2. Characterization of peripheral inflammation in liver tissues of mice received multi-dose administration of AgNPs. **Mice were injected with 25 µg AgNP-10, AgNP-75 or AgNP-110 on Day 1, 4 and 10. The tissue sample of liver was collected on the day 7 after the last injection.** The upper row was the percentage of monocytes or neutrophils in peripheral inflammation of the liver. The lower row was the representative images of liver for quantification of infiltrated cells. Green arrow points to monocytes, red arrow points to neutrophils. The scale bar represents 10 μm.


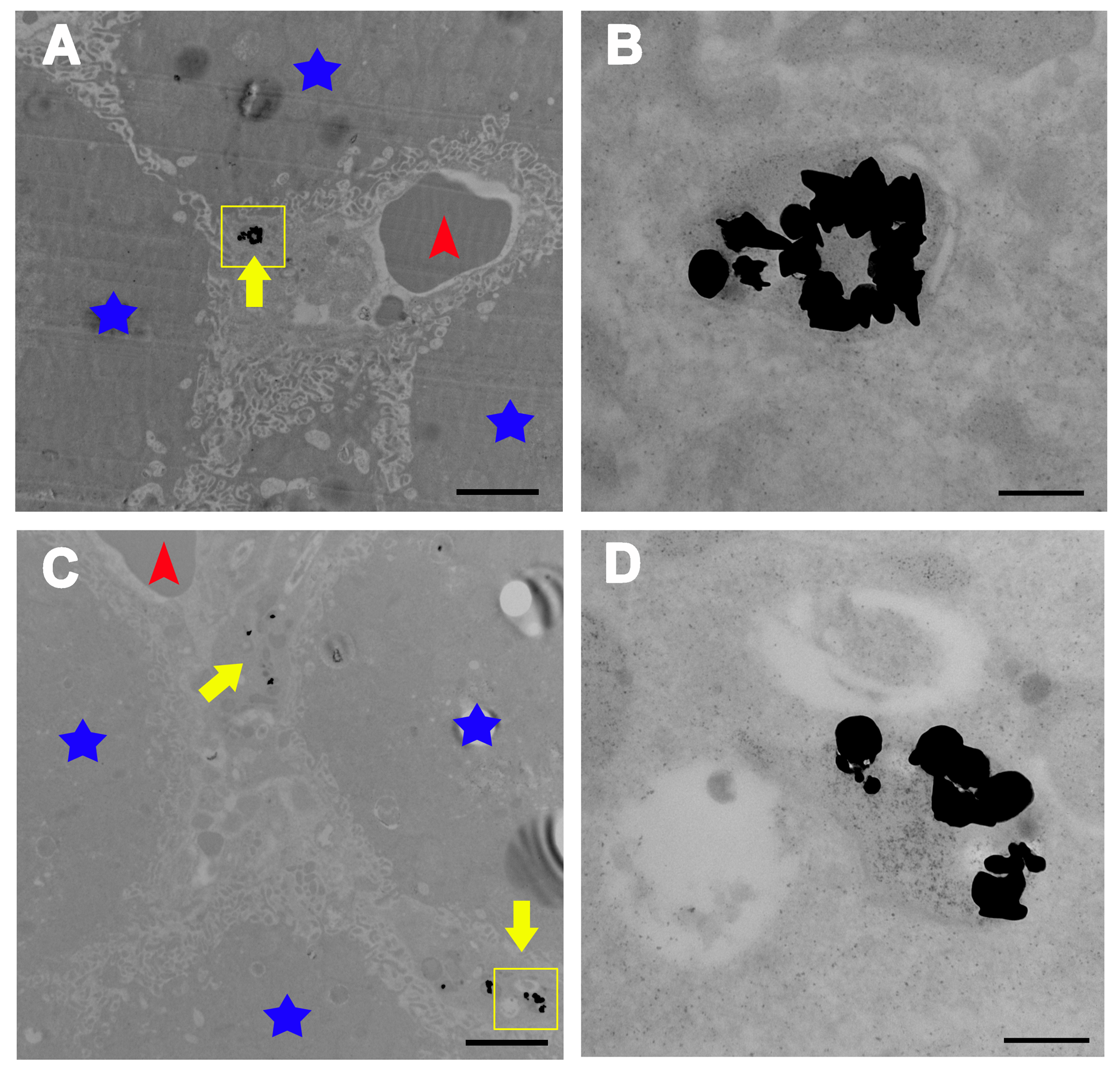


Supplementary Figure S3. Localization of AgNP-110 in the liver of mice received intravenous administration of AgNP-110. A and C: TEM observation of liver after injection of AgNPs for 1 h and 24 h, the scale bar represents 2 μm. B and D: magnification of A and C, the scale bar represents 200 nm. Red arrow head points one red cell in the microvessel, blue star points hepatic cells, and yellow arrow points AgNPs location.


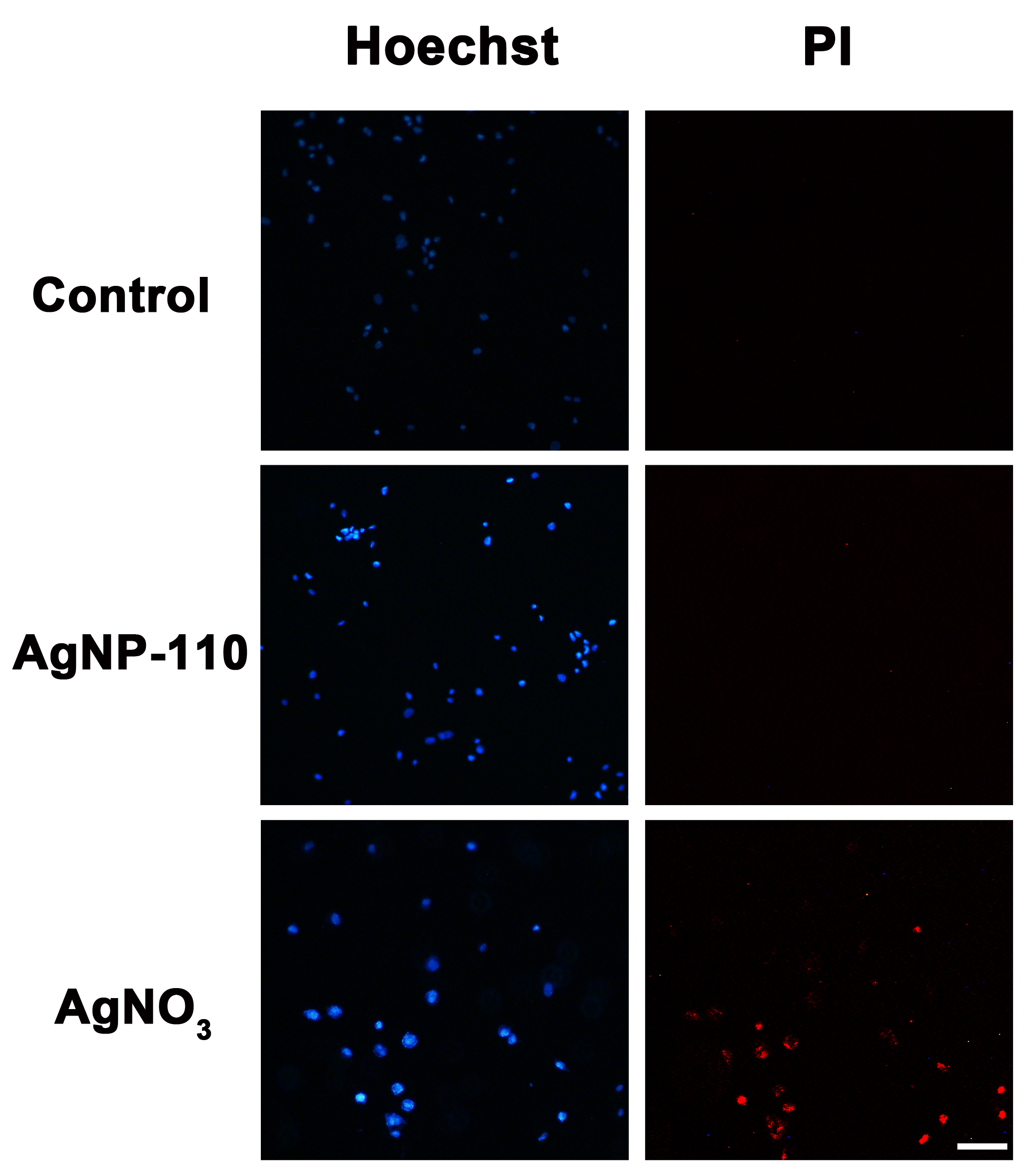


**Supplementary Figure S4. Representative fluorescent images of cells stained by** **Hoechst/PI after exposure to AgNP-110 or** **AgNO_3_. Cells were incubated AgNP-110 at 10 µg/mL for 40 min or AgNO_3_ at 10 µg/mL of Ag for 20 min. The scale bar represents 100 μm.**
